# Associations between visual perception accuracy and confidence in a dopaminergic manipulation study

**DOI:** 10.3389/fpsyg.2015.00414

**Published:** 2015-04-16

**Authors:** Christina Andreou, Vasilis P. Bozikas, Thies Luedtke, Steffen Moritz

**Affiliations:** ^1^Department of Psychiatry and Psychotherapy, University Medical Center Hamburg-EppendorfHamburg, Germany; ^2^1^*st*^ Department of Psychiatry, Aristotle University of ThessalonikiThessaloniki, Greece

**Keywords:** dopamine, haloperidol, drug-challenge, reasoning biases, sensory perception

## Abstract

Delusions are defined as fixed erroneous beliefs that are based on misinterpretation of events or perception, and cannot be corrected by argumentation to the opposite. Cognitive theories of delusions regard this symptom as resulting from specific distorted thinking styles that lead to biased integration and interpretation of perceived stimuli (i.e., reasoning biases). In previous studies, we were able to show that one of these reasoning biases, overconfidence in errors, can be modulated by drugs that act on the dopamine system, a major neurotransmitter system implicated in the pathogenesis of delusions and other psychotic symptoms. Another processing domain suggested to involve the dopamine system and to be abnormal in psychotic disorders is sensory perception. The present study aimed to investigate whether (lower-order) sensory perception and (higher-order) overconfidence in errors are similarly affected by dopaminergic modulation in healthy subjects. Thirty-four healthy individuals were assessed upon administration of l-dopa, placebo, or haloperidol within a randomized, double-blind, cross-over design. Variables of interest were hits and false alarms in an illusory perception paradigm requiring speeded detection of pictures over a noisy background, and subjective confidence ratings for correct and incorrect responses. There was a significant linear increase of false alarm rates from haloperidol to placebo to l-dopa, whereas hit rates were not affected by dopaminergic manipulation. As hypothesized, confidence in error responses was significantly higher with l-dopa compared to placebo. Moreover, confidence in erroneous responses significantly correlated with false alarm rates. These findings suggest that overconfidence in errors and aberrant sensory processing might be both interdependent and related to dopaminergic transmission abnormalities in patients with psychosis.

## Introduction

It has long been proposed that conscious perception is not the mere result of sensory stimulation, but reflects an interaction between sensation and previous experience (Von Helmholtz, 1867). More recent accounts regard perception as a process of Bayesian inference, whereby bottom–up processing of sensory signals is combined with the top–down influence of internally generated predictions; the latter are based on models (acquired through previous experience) of how sensations occur (Friston, 2005). Dopaminergic neurotransmission plays an important role across several steps of this process: At the lowest processing level, it is thought to promote salient stimulus detection by regulating thalamo-cortical interactions (Happel et al., 2014). Moreover, it plays a major role in integrating endogenous predictions with sensory input by encoding prediction errors (Kapur, 2003) with regard to the reward value or expectedness of a stimulus (Bromberg-Martin et al., 2010).

The above are relevant in the context of neurobiological theories of psychosis. The most prominent and enduring account for the emergence of psychotic symptoms such as delusions and hallucinations postulates that these result from a hyperdopaminergic state in the brain (Meltzer and Stahl, 1976). Although the dopamine hypothesis has undergone several refinements over the years, its major premise has remained unchanged (Howes and Kapur, 2009). In fact, all currently licensed antipsychotic drugs have as a common denominator their ability to block dopamine D2 receptors (Howes and Kapur, 2009). Based on the role of dopaminergic transmission in perceptual processes, it has been suggested that an excess of dopamine leads to aberrant stimulus salience by affecting (a) the signal-to-noise ratio of sensory processing, such that noise is perceived as a meaningful signal (Corlett et al., 2010); and/or (b) the prediction error signal, such that neutral or innocuous stimuli are perceived as bearing significance (Kapur, 2003). The attempt of the cognitive system to make sense of such aberrant experiences leads to the emergence of delusions (Kapur, 2003; Fletcher and Frith, 2009).

However, the aberrant salience model does not explain the incorrigibility and high conviction, with which delusional ideas are adopted; it has been pointed out (Lincoln, 2007; Langdon et al., 2010) that abnormal phenomena such as depersonalization, ‘deja-vu’ or telepathy are experienced by healthy individuals as well, but are not uncritically accepted as being true. Cognitive theories of delusions approach thus the symptom from this perspective. In this framework, delusions are regarded as resulting from specific disruptions in the normal processes for belief generation and evaluation (Langdon et al., 2010). Such ‘metacognitive’ disruptions, subsumed under the term ‘reasoning biases,’ correspond to thinking styles that lead to a distorted integration and interpretation of perceived stimuli (Bell et al., 2006; Freeman, 2007). Several such biases have been consistently shown to be associated with delusions, such as jumping-to-conclusions (i.e., a tendency to draw inferences based on limited evidence; Garety and Freeman, 2013), increased confidence in false judgments (Moritz et al., 2008), and a bias against disconfirmatory evidence (Woodward et al., 2006). Importantly, these biases have been confirmed for non-delusional material, precluding tautological inferences.

It is not yet clear how reasoning biases relate to aberrant salience. One account postulates that the two abnormalities represent distinct steps in delusion formation (Langdon et al., 2010), whereas others argue that reasoning biases might directly result from aberrant stimulus salience (Fletcher and Frith, 2009; Corlett et al., 2010). However, there is little empirical support in favor of either assumption. To our knowledge, only one recent study (Schmack et al., 2013) investigated the association between lower-order perceptual processing, higher-level predictions and delusion proneness. The authors reported that higher delusional conviction was associated with more unstable (low-level) visual perception, but also with a stronger top–down influence of beliefs on perception. The latter was accompanied by increased functional connectivity between frontal and primary sensory areas, confirming that aberrant lower-level sensory processing might lead to an enhanced top–down influence of beliefs on perception (Schmack et al., 2013).

Previous studies by our group have approached the issue from a different point of view. As delusions are thought to result from abnormally increased dopaminergic activity, we investigated the effect of dopaminergic manipulation on delusion-associated reasoning biases. We observed that dopamine antagonists (i.e., antipsychotics) reduced overconfidence in errors in healthy participants (Andreou et al., 2014a), and increased subjective doubt in patients with psychotic and other psychiatric disorders (Moritz et al., 2013). Thus, the same neurochemical abnormality, aberrant dopamine activity, might be responsible for abnormalities in both lower-level (sensory perception and salience) and higher-level processing (overconfidence in errors). The present study aimed to investigate this issue, by assessing the effects of dopamine agonists and antagonists on both accuracy and subjective confidence during a visual detection task. Given the postulated effects of dopamine on sensory perception, it was expected that administration of a dopamine agonist would lead to (a) increased stimulus detection salience, reflected in an increased rate of successful detections (hits) but also an increased rate of false alarms, and (b) increased confidence in error responses. It was further expected that detection performance would be significantly correlated with error overconfidence.

## Materials and Methods

The present study was part of a larger project investigating the effects of dopaminergic agonists and antagonists on cognitive functions associated with psychotic symptoms, such as semantic priming and reasoning biases.

### Participants and Design

Participants were 34 healthy individuals aged 18–40 years (18 male, mean age 26.4 ± 4.65) recruited through postings on university recruitment sites. The sample size was calculated based on effect sizes regarding dopaminergic manipulation of reasoning biases observed in a previous study by our group (Andreou et al., 2014a), with which there was no participant overlap. Exclusion criteria were any past or current psychiatric or neurological disorder (including substance use disorders), a history of schizophrenia or bipolar disorder in a first-degree relative, a history of cranio-cerebral trauma, arterial hypertension, cardiologic or serious medical conditions, pregnancy, or treatment with any psychotropic or other drugs. Eligibility for the study was confirmed by means of an interview. The study was approved by the Ethics Committee of the Medical Association Hamburg (Germany), and was performed in accordance with the ethical standards described in the Declaration of Helsinki. All participants provided written informed consent before participating in the study.

In order to assess the effects of dopaminergic agents on reasoning biases, a randomized, double-blind, three-way cross-over design was used (Andreou et al., 2014a,b). In three successive visits, participants were administered either 100 mg L-Dopa and 25 mg benserazide (Madopar®), 2 mg haloperidol (Haldol®), or placebo, in randomized order and under double-blind conditions (see Andreou et al., 2014a for the dose selection rationale). The three visits were separated by at least 7 days, in order to allow a complete wash-out of the drug with the longer half-time (haloperidol Hardman et al., 2001). In order to compensate for the different Tmax (time to reach maximal serum concentration) of haloperidol and L-dopa (Hardman et al., 2001), a double-dummy design was implemented (see Andreou et al., 2014a). The testing session began thus at the time of maximal serum concentration of each drug, and lasted 60 min at the maximum. Subjective psychological, somatic and motor (adverse) effects of the drugs were assessed through ratings on a 42-item Likert scale at baseline, at the time of ingestion of the second capsule and after the end of the testing session; moreover, blood pressure, and pulse were measured at 30-min intervals. In order to assess the success of the blinding procedure, participants were asked to guess which substance they had received at the end of each session. The d2-test, a letter-cancelation task with well documented validity and excellent test–retest reliability (Brickenkamp, 1981), was also administered at each session to rule out performance differences due to non-specific effects of the drugs on attention.

Psychotic experiences were assessed with the Community Assessment of Psychic Experiences-42 (CAPEs-42) Scale, a 42-item self-report questionnaire that yields scores for positive, negative, and depressive symptoms (Konings et al., 2006). The scale was completed at the end of each testing session; small adjustments to item wording were made, such that the reference time period corresponded to the duration of the session.

### Perceptual Confidence Task

**FIGURE 1:**
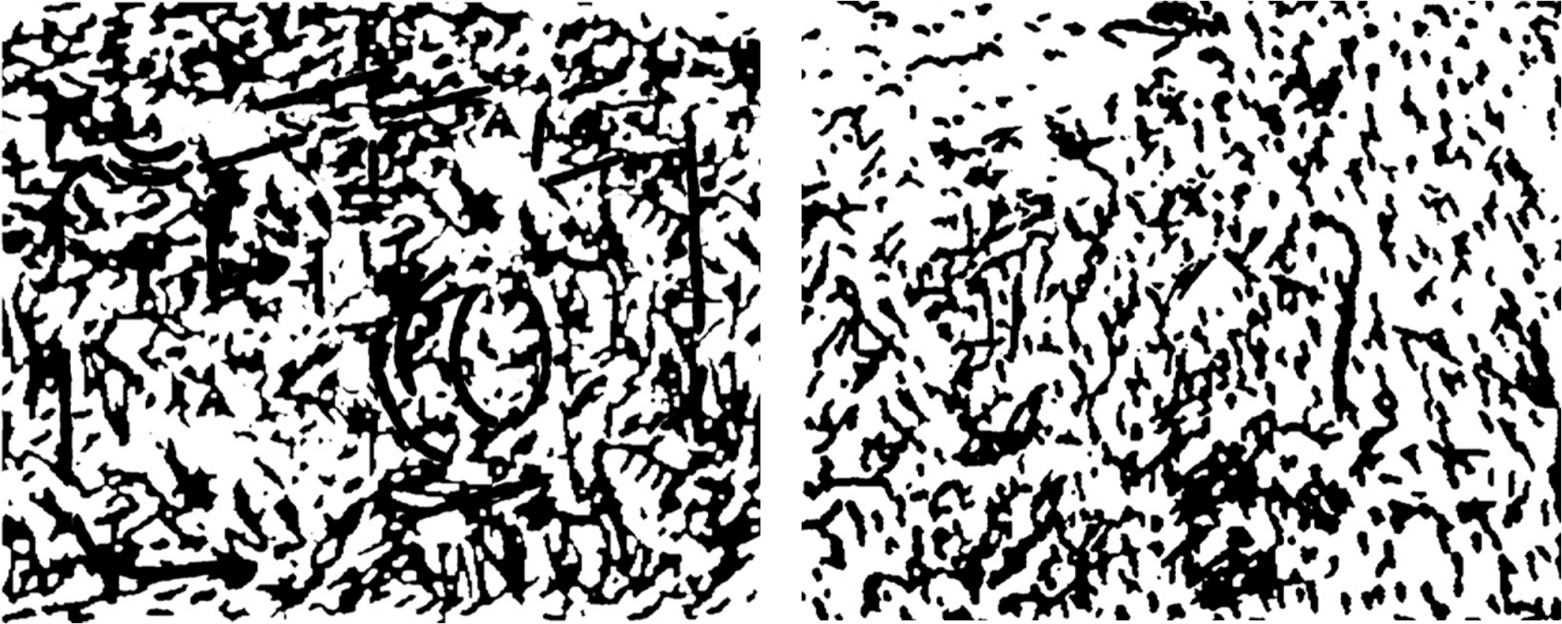
**Example of stimuli used in the visual perception task: the picture on the left contains an embedded image (a photographic camera); the picture on the right consists only of noise**.

A computerized variant of the Snowy Pictures Task (Whitson and Galinsky, 2008) was used to assess visual perception accuracy and confidence. The experiment was presented using E-Prime® 2.0 (Schneider et al., 2002). Participants were presented “snowy” (noisy) pictures, some of which contained a hidden object (see **Figure 1** for stimulus examples). The task was administered as a speeded response task: after a fixation of 500 ms, each picture was shown for 1 s on a computer screen, and participants were instructed to press a key within a time window of 2 s from stimulus onset, if they thought that an object was hidden in the picture. Participants were instructed to respond as quickly and as accurately as possible. After responding, participants rated their subjective confidence in their response on a scale from 0–100% (in steps of 12.5%). For the present study, three parallel versions were developed using stimuli constructed in a similar manner as those of the original task (Whitson and Galinsky, 2008). The three versions were matched for stimulus difficulty (established in a pretest) and luminance (*p* > 0.75). Each parallel version comprised 37 pictures, of which 16 contained an object and 21 did not. Variables of interest were hit and false alarm rate, as well as mean subjective confidence for correct and incorrect responses.

### Statistical Analyses

Prior to analysis, we removed all misses and false alarm trials with a confidence rating of zero (indicating certainty to have made an error), assuming that they, respectively, reflected failed (too slow) and accidental key presses. This resulted in exclusion of 1.2% of trials (4.2% of errors). Moreover, sessions with hit and/or false alarm rates exceeding 2 SDs from the group mean (corresponding to a hit rate<50% and/or a false alarm rate of >70%) were regarded as outliers and excluded from analysis (*n* = 9 sessions from eight participants). Finally, eight further sessions were removed from error confidence analyses only – five sessions (from four participants) because confidence means were based on less than five errors, an a priori determined criterion set to avoid unrepresentative means; and all three sessions of one participant who indicated a level of 100% confidence in all trials.

As the above procedure resulted in missing data, statistical analyses were conducted using linear mixed models. In contrast to repeated-measures ANOVAs, linear mixed models can be successfully estimated even in the case of missing data (Field, 2013, p. 818); additionally, they are better suited to model interindividual variability, as they can accommodate departures from the assumptions of homogeneity of regression slopes and independence (Gueorguieva and Krystal, 2004; Field, 2013, p. 818). Separate linear mixed models were estimated for each of the following dependent variables: hit rate, false alarm rate, subjective confidence in correct responses, subjective confidence for incorrect responses, and reaction time for key presses. In all cases, ingested substance (haloperidol, L-dopa, or placebo) was included as the only fixed-effects, repeated-measures predictor, while participant ID was modeled as a random-effects predictor. Session (first, second, or third) and its interaction with substance were initially also included in these models in order to check for practice effects, but these predictors were removed again as they were not significant for any of the above dependent variables (all *p* > 0.35). The optimal covariance structure for each linear mixed model was determined using goodness-of-fit criteria (Akaike’s Information Criterion; AIC). Significant substance effects were followed-up with *post hoc* pairwise comparisons (least significant difference method), and additionally with polynomial contrasts to investigate linear and quadratic trends in performance.

Similar analyses were conducted for the positive, negative, and depression subscale of the CAPE. However, the effect of session was significant in all cases, such that the linear mixed models included both session and ingested substance as fixed-effects predictors.

## Results

There were no significant differences among the three substances in d2-scores, nor in adverse effects (no missing data; repeated-measures ANOVAs, all *p* > 0.14). There were no drop-outs and no premature session terminations due to adverse effects. There was also no association between ingested and guessed substance [χ^2^ = 8.14, *p* = 0.23]. Participant scores on all variables of interest are presented in **Table 1**. For descriptive purposes, reaction times and confidence ratings per substance and response type (hits, misses, correct rejections, false alarms) are presented on **Table 2**.

**Table 1:** Mean and SD of detection performance, subjective confidence and subjective psychotic experiences after administration of haloperidol, l-dopa, and placebo.

	Haloperidol		Placebo		L-dopa	
	Mean	SD	Mean	SD	Mean	SD
Hit rate (%)	78.26	10.2	80.36	12.4	80.31	14.0
False alarm rate (%)	22.75	14.2	29.64	17.6	30.94	20.5
Confidence rating – correct responses	80.81	12.0	78.29	11.2	79.84	12.1
Confidence rating – incorrect responses	67.51	17.7	66.49	12.4	71.70	13.6
CAPE positive score	21.73	3.4	21.91	3.6	22.44	4.6
CAPE negative score	18.48	4.1	18.97	6.5	18.92	6.3
CAPE depression score	9.53	2.5	10.03	3.4	10.00	2.7

**Table 2:** Mean and SD for reaction times and confidence ratings per substance and response type.

	Haloperidol				Placebo				L-dopa			
	Reaction time		Confidence		Reaction time		Confidence		Reaction time		Confidence	
	Mean	SD	Mean	SD	Mean	SD	Mean	SD	Mean	SD	Mean	SD
False alarms	732.06	97.5	69.27	22.6	728.22	129.5	69.60	14.4	734.96	181.4	73.67	14.8
Hits	681.47	89.0	87.47	10.2	681.28	103.3	85.03	9.2	677.46	112.2	86.72	9.0
Correct rejections^∗^	–	–	75.67	16.0	–	–	71.66	15.3	–	–	72.54	18.0
Misses^∗^	–	–	65.88	19.9	–	–	63.34	18.4	–	–	62.57	22.6

^∗^Participants withheld responses, therefore reaction times are not available.

Hit rate did not significantly differ between substances [*F*(2,32.48) = 1.022, *p* = 0.37], but there was a significant main effect of substance with respect to the false alarm rate [*F*(2,29.67) = 3.688, *p* = 0.04]. Polynomial contrasts revealed a significant linear trend (*t* = 2.419, *p* = 0.02) indicating an increase in false alarm rates from haloperidol to placebo to L-dopa, while the quadratic trend was not significant. *Post hoc* pairwise comparisons were significant for the comparison of false alarm rate under haloperidol compared to placebo (*p* = 0.01) and to L-dopa (*p* = 0.02), while placebo and L-dopa did not significantly differ from each other (*p* = 0.71).

With regard to subjective confidence for incorrect responses, there was a trend toward an effect of substance [*F*(2, 22.66) = 3.013, *p* = 0.07]. Both linear and quadratic effects missed significance. Pairwise comparisons indicated that confidence for error responses was higher with L-dopa compared to placebo (*p* = 0.03), whereas there were no differences between haloperidol and either placebo (*p* = 0.54) or L-dopa (*p* = 0.15). Subjective confidence for correct responses did not significantly differ across substances [*F*(2,24.54) = 1.684, *p* = 0.21].

The ingested substance had a significant effect of CAPE positive symptoms [*F*(2,26.83) = 3.546, *p* = 0.04], with participants scoring trend-wise higher with L-dopa compared to placebo (*p* = 0.06) and haloperidol (*p* = 0.08), which did not differ from each other (*p* = 0.82).

### Subsidiary Analyses

In accordance with our hypothesis, both false alarm rate and (to a lesser extent) subjective confidence in error responses were influenced by dopaminergic modulation. Therefore, we investigated the associations between variables. In order to account for multiple values obtained with different substances in each participant, we again conducted a linear mixed model, in which substance was modeled as a repeated effect, and participant ID as a random effects variable. False alarm rate was the dependent variable, while confidence for incorrect responses was entered as a continuous predictor in the model. The result was significant [*F*(1,78.87) = 4.11, *p* < 0.05], with a positive *b* = 0.0027 (β = 0.224) indicating that increased confidence for incorrect responses was associated with increased false alarm rates.

## Discussion

The present study aimed to investigate how single-dose administration of dopaminergic agonists and antagonists affects brain functions associated with delusions across two different processing levels – perceptual (lower-order) and metacognitive (higher-order). Dopaminergic manipulation significantly affected false-alarm rate on a visual perception task, with a linear trend indicating a gradual increase of false alarms from haloperidol to placebo to L-dopa. An effect of dopaminergic agents was also evident, although to a lesser extent, for overconfidence in errors. Critically, perceptual and metacognitive performance were significantly correlated with each other, suggesting that the two levels of processing might be interdependent.

It has been suggested that conscious perception is based on internal representations of the statistical behavior of the own sensory/perceptual systems; based on these representations, a ‘response criterion’ is set that determines the signal-to-noise ratio threshold, at which the stimulus is perceived as a meaningful signal (Lau, 2008). In this framework, increased dopaminergic activity would be expected to lead to a more liberal response criterion by affecting these internal representations – in other words, by affecting endogenous predictions and the prediction error signal (Fletcher and Frith, 2009; Corlett et al., 2010). In turn, this should result in an increased rate of both successful stimulus detections (hits) and false alarms. For example, a recent study on speeded visual word recognition reporting higher accuracy rates with a dopaminergic agonist compared to placebo (Lou et al., 2011). In clinical populations, individuals that experience psychotic symptoms demonstrate increased false alarm rates in various perceptual paradigms (summarized in Tsakanikos and Reed, 2005), while dopaminergic hypoactivity in Parkinson’s disease has been associated with reduced stimulus detection rates (Horowitz et al., 2006).

In the present study, our hypothesis regarding stimulus detection accuracy was only partially confirmed: dopaminergic manipulation had an effect only on false-alarm rate, while hit rate remained unaffected. This finding can be interpreted in terms of signal detection theory. According to the latter, the decision whether a stimulus is present or not is based on the strength of an internal decision signal, which is assumed to have a Gaussian distribution and to have a higher mean when a stimulus is present than when it is absent. As explained above, the cut-off value for discriminating a stimulus from noise (*response criterion*) is based on internal representations their respective signal distributions, derived from previous experience (Lau, 2008). According to this framework, an increase in false alarms that is not accompanied by increased hit rates would suggest that the distribution of the noise signal is shifted toward higher values, i.e., closer to the “stimulus present” distribution, while the response criterion remains unaffected. However, there are also other explanations for the dissociation of results regarding false alarms and hits in the present study. For example, this finding may have been due to ceiling effects, as overall hit rates in the placebo condition were quite high in the present sample. In support of this interpretation are findings of a recent study (Lou et al., 2011), in which differences in accuracy rates between placebo and the dopaminergic agonist pergolide were apparent only in the more difficult versions of the administered task. An alternative explanation for the negative finding regarding hit rates is that the effects of dopaminergic agents on perceptual detection performance might be dependent on additional factors that were not captured by our study design: for example, a previous study (Krummenacher et al., 2010) reported a complex pattern of L-dopa effects on signal detection performance depending on the presence or absence of schizotypal traits in participants.

With respect to our second hypothesis, our results are largely in line with a previous study by our group, in which single-dose administration of dopaminergic agonists and antagonists significantly affected confidence in memory errors in healthy subjects (Andreou et al., 2014a). Moreover, the observed increase in error confidence under L-dopa parallels previous findings by our group in patients with schizophrenia (Moritz et al., 2014b) and individuals scoring high on schizotypy (Moritz et al., 2014a) using a similar visual perception task. Our findings are also consistent with early observations (Moritz et al., 2003, 2008) of a negative correlation between antipsychotic medication dose and overconfidence in errors in patients with schizophrenia. More importantly, confidence in errors was correlated with false alarm rate in the present study, confirming that deficient processing at the sensory/perceptual level may lead to increased reliance on higher-level predictions, i.e. top–down processing (Corlett et al., 2010; Schmack et al., 2013). This suggests that one single disturbance, aberrant stimulus salience, suffices to account both for the emergence of delusions and their tenacity. However, it should be kept in mind that different reasoning biases are independent from each other (Moritz et al., 2010), and that an association with dopamine could not be confirmed for another prominent delusion-related bias, jumping-to-conclusions (Andreou et al., 2014a; Ermakova et al., 2014). Therefore, it remains to be tested in future studies whether the observed associations between stimulus salience and overconfidence extend to jumping-to-conclusions or other reasoning biases.

A strength of the present study lies in the inclusion of assessments of subjective psychotic experiences; the finding that single-dose administration of haloperidol and L-dopa led to the expected changes in a self-rated scale of psychotic experiences lends credibility to the postulated association between perception, confidence, and delusions. However, it should be noted as a limitation that changes in the dopaminergic system brought about by the psychotic state and its treatment are multiple and complex (Kim et al., 2011; Howes et al., 2012), and are unlikely to be fully approximated by single-dose administration of dopaminergic agents in healthy subjects. Thus, caution is advised when extrapolating findings to patients with schizophrenia, especially since these additionally present complex deficits in perceptual organization, which are associated with disorganized rather than delusional symptoms and are dependent on the glutamate system (Silverstein and Keane, 2011).

In summary, dopaminergic manipulations led to parallel changes in two delusion-associated brain functions, visual stimulus detection and subjective confidence in errors. These findings suggest that overconfidence in errors and aberrant sensory processing might be both interdependent and related to dopaminergic transmission abnormalities in patients with psychosis.

## Conflict of Interest Statement

The authors declare that the research was conducted in the absence of any commercial or financial relationships that could be construed as a potential conflict of interest.
